# Infection of Endothelial Cells with Acinetobacter baumannii Reveals Remodelling of Mitochondrial Protein Complexes

**DOI:** 10.1128/spectrum.05174-22

**Published:** 2023-04-13

**Authors:** Laura Leukert, Manuela Tietgen, Felix F. Krause, Tilman G. Schultze, Dominik C. Fuhrmann, Charline Debruyne, Suzana P. Salcedo, Alexander Visekruna, llka Wittig, Stephan Göttig

**Affiliations:** a Institute for Medical Microbiology and Infection Control, University Hospital, Goethe University, Frankfurt am Main, Germany; b University Center of Competence for Infection Control of the State of Hesse, Frankfurt am Main, Germany; c Institute for Medical Microbiology and Hygiene, Philipps-University, Marburg, Germany; d Institute of Biochemistry I, Faculty of Medicine, Goethe University, Frankfurt am Main, Germany; e Laboratory of Molecular Microbiology and Structural Biochemistry, Centre National de la Recherche Scientifique UMR5086, Université de Lyon, Lyon, France; f Functional Proteomics, Institute of Cardiovascular Physiology, Goethe University, Frankfurt am Main, Germany; Agroscope

**Keywords:** *Acinetobacter baumannii*, infection, host-pathogen interaction, *Galleria mellonella*, virulence genes, mitochondria, international cluster 2 (IC2), oxidative phosphorylation

## Abstract

Acinetobacter baumannii is an antibiotic-resistant, Gram-negative pathogen that causes a multitude of nosocomial infections. However, pathogenicity mechanisms and the host cell response during infection remain unclear. In this study, we determined virulence traits of A. baumannii clinical isolates belonging to the most widely disseminated international clonal lineage, international cluster 2 (IC2), *in vitro* and *in vivo*. Complexome profiling of primary human endothelial cells with A. baumannii revealed that mitochondria, and in particular complexes of the electron transport chain, are important host cell targets. Infection with highly virulent A. baumannii remodelled assembly of mitochondrial protein complexes and led to metabolic adaptation. These were characterized by reduced mitochondrial respiration and glycolysis in contrast to those observed in infection with low-pathogenicity A. baumannii. Perturbation of oxidative phosphorylation, destabilization of mitochondrial ribosomes, and interference with mitochondrial metabolic pathways were identified as important pathogenicity mechanisms. Understanding the interaction of human host cells with the current global A. baumannii clone is the basis to identify novel therapeutic targets.

**IMPORTANCE** Virulence traits of Acinetobacter baumannii isolates of the worldwide most prevalent international clonal lineage, IC2, remain largely unknown. In our study, multidrug-resistant IC2 clinical isolates differed substantially in their virulence potential despite their close genetic relatedness. Our data suggest that, at least for some isolates, mitochondria are important target organelles during infection of primary human endothelial cells. Complexes of the respiratory chain were extensively remodelled after infection with a highly virulent A. baumannii strain, leading to metabolic adaptation characterized by severely reduced respiration and glycolysis. Perturbations of both mitochondrial morphology and mitoribosomes were identified as important pathogenicity mechanisms. Our data might help to further decipher the molecular mechanisms of A. baumannii and host mitochondrial interaction during infection.

## INTRODUCTION

The dissemination of multidrug-resistant Gram-negative bacteria has increased dramatically within the last years and poses a global threat to health care systems. Acinetobacter baumannii is one of the most important pathogens causing severe nosocomial and health care-associated infections. Different clonal lineages of A. baumannii can be divided into at least eight international clusters (ICs) based on their core genome; IC2 is by far the most predominant clone worldwide (1). A. baumannii possesses the outstanding ability to exhibit multidrug-resistant phenotypes by acquisition of mobile antibiotic resistance determinants and to persist under harsh environmental conditions like high salt concentrations or desiccation (2). The first-line therapy to combat infections caused by A. baumannii is the administration of carbapenems (3). However, porin deficiency, modification of penicillin-binding proteins, efflux pump activity, and especially the acquisition of OXA-type carbapenem-hydrolyzing class D β-lactamases lead to carbapenem resistance (4–7). Isolates of the IC2 often harbor OXA-23, which is currently the most prevalent acquired carbapenemase in A. baumannii (1).

A. baumannii possesses different virulence factors that modulate pathogenicity with impact on the host cell response during infection (8–10). For example, the Acinetobacter trimeric autotransporter adhesin Ata and the outer membrane protein OmpA mediate adhesion to the host cell, which is a crucial early step during infection (11, 12). In addition to Ata, the phospholipases D are involved in the invasion by A. baumannii of epithelial and endothelial cells (11, 13). The plasminogen-binding protein CipA leads to the inactivation of the alternative pathway of the complement system, thereby circumventing host defense mechanisms (14). A. baumannii can also modulate host cell signaling, trigger cytotoxicity, and induce apoptosis by concerted action of various virulence factors (11, 15). Apoptosis can be induced by an extrinsic and/or intrinsic pathway. In the extrinsic pathway, the death-inducing signaling complex (DISC) is formed upon ligand binding at the cell surface, leading to activation of caspase-8. The intrinsic pathway is characterized by the release of cytochrome *c* of mitochondria, followed by the formation of the apoptosome and subsequent activation of caspase-9. Both caspases-8 and -9 can activate effector caspases like caspase-3 or caspase-7 (16).

The pathogenicity of A. baumannii is most commonly investigated with epithelial cell lines employing the lab-domesticated strains ATCC 19606^T^ and ATCC 17978, which were isolated >70 years ago. Yet, these strains are not multidrug resistant and are genetically fairly unrelated to any of the worldwide-circulating ICs. As a consequence, little is known about the pathogenicity mechanisms of the current carbapenem-resistant clonal lineages. Furthermore, we recently observed an important role of endothelial cells in host-pathogen interaction during infection with A. baumannii (11, 17). This prompted us to investigate virulence of carbapenem-resistant A. baumannii clinical isolates of the worldwide predominant clonal lineage IC2 *in vivo* and by employing primary human umbilical cord vein endothelial cells (HUVECs).

In this study, we identified mitochondria as an important target organelle during A. baumannii IC2 infection using complexomics. Perturbation of oxidative phosphorylation (OXPHOS), destabilization of mitochondrial ribosomes, and interference with mitochondrial metabolic pathways were identified as important pathogenicity mechanisms.

## RESULTS

### Virulence screening of A. baumannii clinical isolates belonging to the international clonal lineage IC2.

To decipher virulence traits of A. baumannii from the worldwide predominant clonal lineage IC2, 14 carbapenem-resistant, OXA-23-harboring patient isolates were employed (Table 1). Two reference strains, ATCC 19606^T^ and AB_5075, were included (18–20). Antibiotic susceptibility testing revealed high MICs for penicillins, cephalosporins, carbapenems, fluoroquinolones, and aminoglycosides for all IC2 isolates (see Fig. S1 and S2 in the supplemental material) and an extensively drug-resistant (XDR) phenotype (21).

**TABLE 1:** Isolate characteristics

Isolate^*a*^	Specimen	Yr of isolation	IC^*b*^	MIC^*c*^ (mg/L)				
				IMP	TOB	SXT	MIN	COL
AB_1372	Nose swab	2011	2	≥32	1	0.125	0.125	0.5
ATCC 19606^T^	Urine	1948	—	1	8	≥32	1	0.5
AB_1523	Urine	2012	2	≥32	≥256	1	8	1.5
AB_3378	Rectal swab	2016	2	≥32	8	≥32	8	0.125
AB_2597	Tracheal secretion	2015	2	≥32	≥256	≥32	16	0.25
AB_1784	Tracheal secretion	2013	2	≥32	≥256	4	0.5	1
AB_2219	Rectal swab	2014	2	≥32	≥256	1	8	1
AB_4514	Sputum	2017	2	≥32	≥256	≥32	8	0.5
AB_2169	Rectal swab	2014	2	≥32	≥256	1	4	1
AB_5075	Wound swab	2008	1	16	16	≥32	0.125	1.5
AB_1494	Clavicular, shot channel	2012	2	≥32	≥256	1	8	1
AB_4401	Wound swab	2017	2	≥32	≥256	≥32	16	0.5
AB_3038	Rectal swab	2016	2	≥32	≥256	1	32	1
AB_705	Rectal swab	2007	2	≥32	64	≥32	8	0.5
AB_1945	Rectal swab	2014	2	≥32	≥256	≥32	8	1
AB_2778	Rectal swab	2015	2	≥32	≥256	≥32	16	2

a ATCC 19606^T^ and AB_5075 were used as reference strains (18–20).

b IC, international cluster. —, unclustered.

c IMP, imipenem; TOB, tobramycin; SXT, trimethoprim-sulfamethoxazole; MIN, minocycline; COL, colistin. All clinical isolates were resistant to meropenem, ciprofloxacin, and levofloxacin using EUCAST breakpoints.

**FIG 1:**
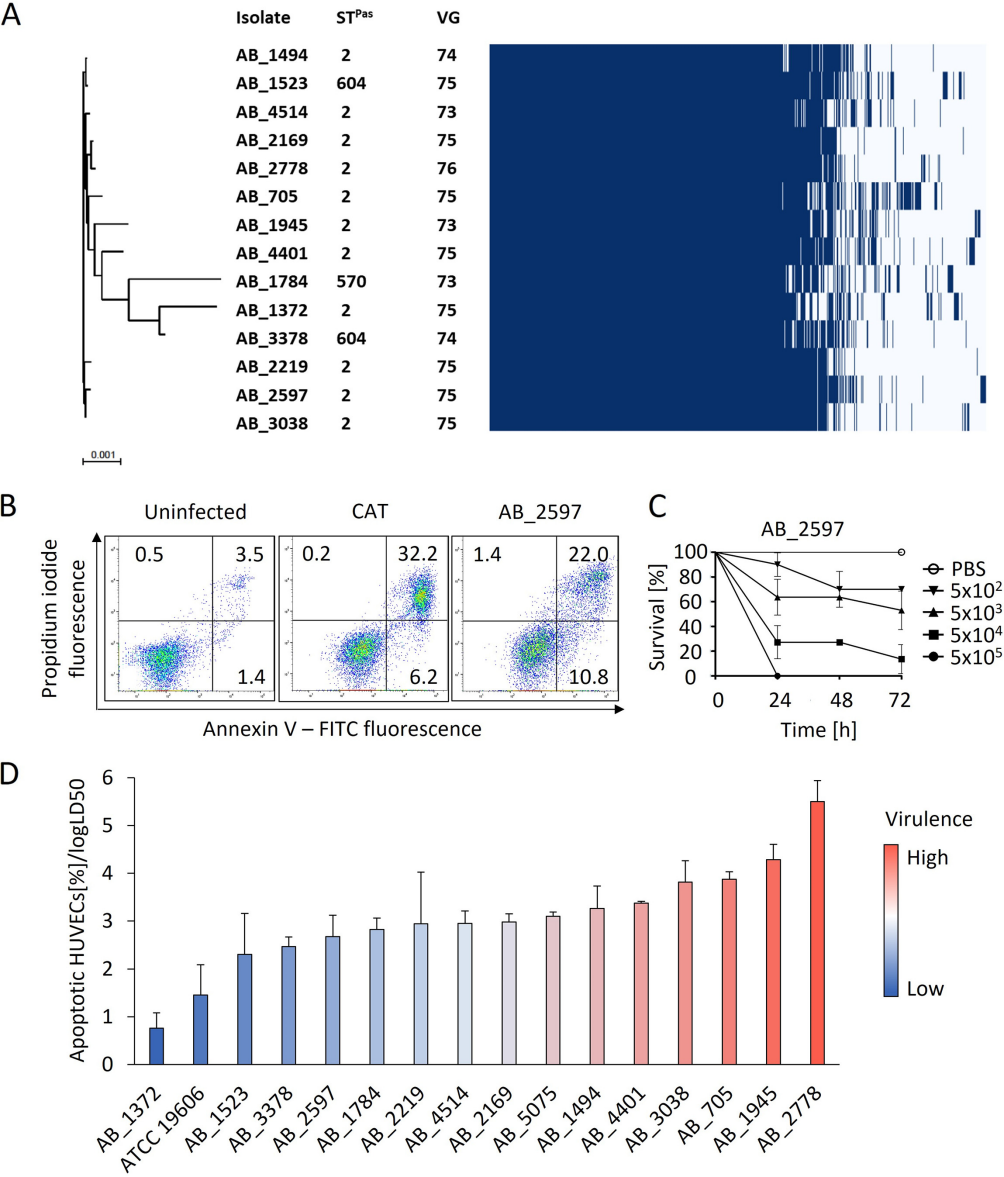
Genetic relatedness and virulence of clinical A. baumannii IC2 isolates. (A) Phylogenetic tree of clinical A. baumannii isolates from IC2 harboring OXA-23 and gene presence-absence matrix with 5,191 gene clusters. ST^Pas^, sequence types according to MLST Pasteur scheme; VG, number of virulence genes. The scale bar indicates nucleotide substitutions per site. (B) Representative flow cytometry plots for cell death quantification showing fluorescence intensities of propidium iodide and annexin V in HUVECs either left uninfected, treated with the topoisomerase I inhibitor camptothecin (CAT), or infected with AB_2597. HUVECs were infected with A. baumannii isolates, stained with propidium iodide and annexin V-FITC, and analyzed by flow cytometry. The gates indicate different stages of cell death: upper left, necrotic cells; upper right, late apoptotic cells; lower right, early apoptotic cells; and lower left, viable cells. Values show mean percentage of the parental cell population. (C) Representative time-kill curve of G. mellonella larvae infected with AB_2597. Larvae were injected with the indicated CFU of A. baumannii isolates from IC2 and the survival was monitored for 72 h. Values are means ± 95% confidence intervals (*n* = 3). (D) Virulence of isolates as determined by the ratio of apoptotic cells relative to the log LD_50_ in the G. mellonella *in vivo* infection model. Statistical analysis was performed via calculating Gauss errors and error propagation (*n* = 3).

Whole-genome sequencing was applied to determine genetic relatedness and presence of virulence genes. Pan-genome analysis of the clinical isolates revealed an extensive core genome consisting of 3,067 genes (Fig. 1A). Pairwise single nucleotide polymorphism (SNP) distance analysis based on the core genome of all IC2 isolates demonstrated that AB_1784 showed the lowest similarity to the other isolates (11,129 single nucleotide variants [SNVs] compared to AB_1372), while the isolates with the closest phylogenetic relatedness differed only by 200 SNVs (AB_1523 and AB_1494). Three different sequence types (STs) were identified: ST2 was the most frequent (11 isolates). ST604 was represented by two isolates (AB_1523 and AB_3378), and ST570 was represented by a single isolate (AB_1784). ST604 and ST570 are single locus variants of ST2. The numbers of virulence genes in all clinical isolates were similar and ranged from 73 to 76 per isolate (Fig. 1A).

The virulence of the clinical isolates was determined *in vitro* by measuring apoptosis in infected HUVECs and *in vivo* using the Galleria mellonella infection model (Fig. 1B to D). Apoptosis was determined by annexin V/propidium iodide (PI) staining and subsequent flow cytometric analysis (Fig. 1B). Induction of apoptosis in infected HUVECs ranged from 22.58% (AB_2778) to 4.59% (AB_1372) of the total cell population (Table S1). Virulence *in vivo* was determined by monitoring survival of infected larvae for 72 h and calculation of the median lethal dose (LD_50_) from time-kill curves (Fig. 1C). Log LD_50_ values varied between 3.85 (AB_1784) and 6.05 (AB_1372) (Table S1). In order to quantify virulence from both assays and to determine the isolates with the lowest and highest virulence, we calculated the ratio of apoptotic HUVECs and log LD_50_. AB_1372 was the least virulent (0.76 ± 0.33) and AB_2778 the most virulent isolate (5.50 ± 0.44) (Fig. 1D). Reference strain AB_5075, previously described as being highly pathogenic (19), was more virulent than ATCC 19606^T^, which was the second least virulent strain overall.

Although the 14 clinical IC2 isolates shared an extensive core genome, virulence differed substantially between the isolates and did not correlate with the presence of virulence genes (Fig. S3). To decipher virulence traits of IC2 isolates, we selected the most virulent isolate, AB_2778, and the least virulent isolate, AB_1372, for further analysis.

### Analysis of virulence traits of AB_1372 and AB_2778.

**FIG 2:**
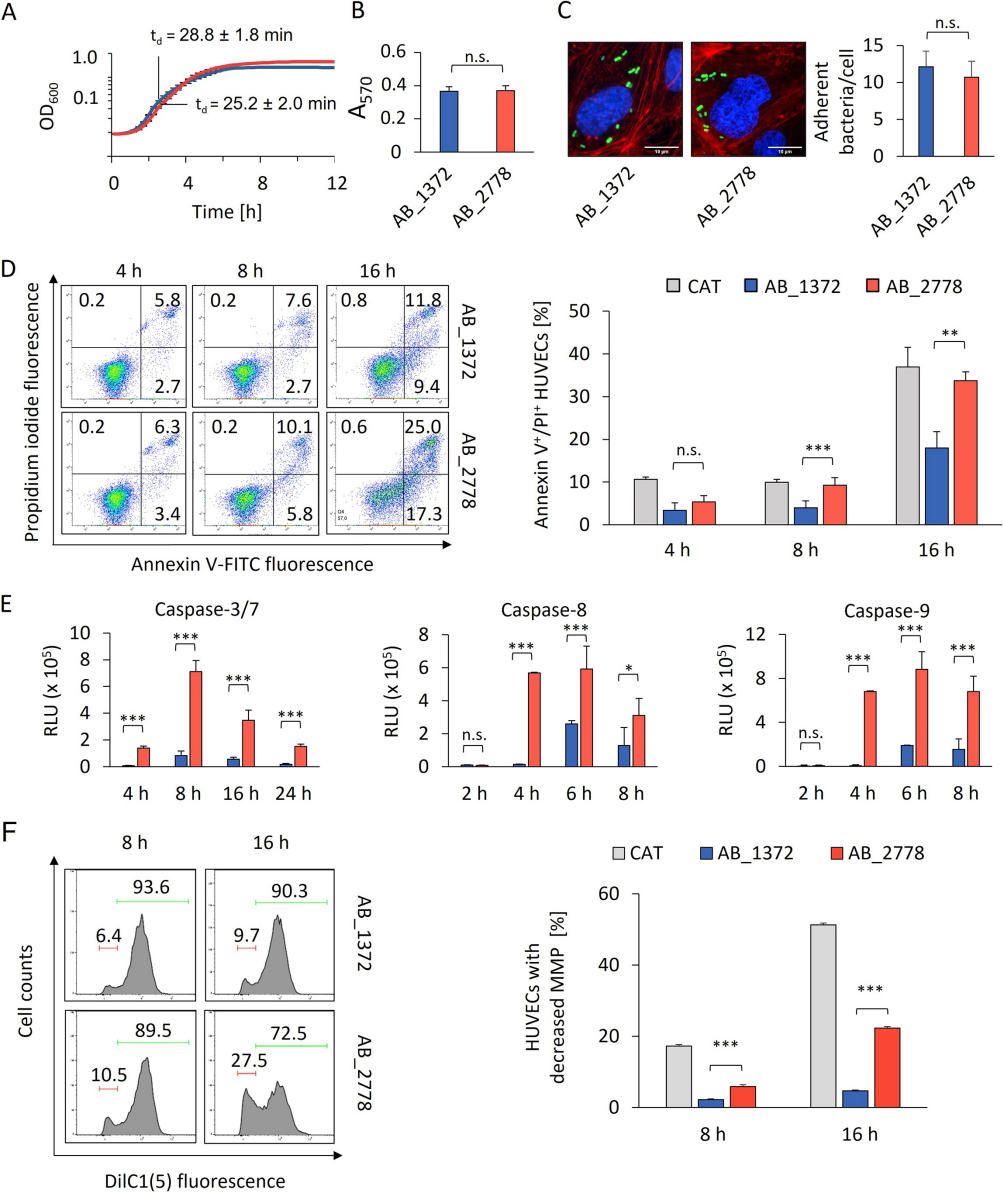
Virulence traits of AB_2778 and AB_1372. (A) Growth analysis of AB_1372 and AB_2778. (B) Biofilm formation of AB_1372 and AB_2778 was quantified by crystal violet staining. (C) Adhesion of AB_1372 and AB_2778 to HUVECs. Fluorescence microscopy of infected HUVECs shows cytoskeleton (red, TRITC-phalloidin), DNA (blue, DAPI), and AB_1372 and AB_2778 (green, carboxyfluorescein succinimidyl ester [CFSE]). (D) Analysis of induction of apoptosis in HUVECs upon infection by propidium iodide/annexin V-FITC staining and flow cytometry. (E) Caspase activation by AB_1372 and AB_2778 in HUVECs as determined by the fluorometric caspase-Glo assay. Values are specified in relative luminescence units (RLU). (F) Analysis of mitochondrial membrane depolarization of HUVECs infected with AB_1372 or AB_2778 for the indicated times. HUVECs were stained with the mitochondrial membrane potential (MMP)-dependent dye DilC1(5). (Left) In the representative fluorescence-activated cell sorting (FACS) plots, the red bars indicate the percentage of HUVECs with mitochondrial membrane depolarization. The green bars indicate the percentage of HUVECs with a stable MMP. (Right) Percentages of cells with depolarized mitochondrial membrane are shown. All data are derived from at least three independent experiments. Values are means ± SD. Statistical analysis was performed by a two-tailed *t* test. *, *P < *0.05; **, *P < *0.01; ***, *P < *0.005 (A to F). OD, optical density; n.s., not significant.

A. baumannii has developed several strategies to initiate and establish an infection in the host, which demands multiple pathogenicity mechanisms. To characterize and compare the virulence traits of AB_1372 and AB_2778, a genome comparison was carried out and assays were performed to evaluate bacterial fitness, biofilm formation, adhesion to HUVECs, and induction of apoptosis (Fig. 2 and Fig. S4).

The growth kinetics of AB_1372 and AB_2778 were similar, suggesting no difference in bacterial fitness (Fig. 2A). Likewise, evaluation of biofilm formation and adhesion to HUVECs revealed no difference, indicating that the virulent phenotype of AB_2778 is not due to increased biofilm formation or adherence (Fig. 2B and C). Next, induction of cell death in HUVECs after infection was evaluated by annexin V/PI staining. At 8 and 16 h postinfection (hpi), apoptosis rates were significantly higher for AB_2778 than for AB_1372 (Fig. 2D). Initiation of apoptosis is induced by proteolytic activation of cysteine-aspartate-specific proteases (caspases) via the extrinsic and intrinsic pathways. Caspases-8 and -9 are the initiator caspases of the extrinsic and intrinsic pathways, respectively. Upon infection of HUVECs with AB_2778, activities of both initiator caspases were higher after 2 hpi than for infection with AB_1372, with maximum values at 6 hpi (Fig. 2E). The activities of the effector caspases-3/7, which act downstream of caspase-8/9, peaked at 8 hpi and were always higher for AB_2778. While caspase-8 is activated by extracellular stimuli via death receptor-ligand binding, caspase-9 is activated through mitochondrial disintegration and cytochrome *c* release. Since mitochondria are important targets for bacteria and other pathogens (22, 23), the impact of A. baumannii on mitochondrial integrity, depolarization of the mitochondrial membrane of infected HUVECs was measured via DilC1(5) staining (Fig. 2F). At 8 hpi, the percentage of HUVECs infected with AB_2778 (5.9%) with decreased mitochondrial membrane potential (MMP) was significantly higher than that of HUVECs infected with AB_1372 (2.2%). For AB_2778, the loss of MMP increased to 22.3% at 16 hpi, suggesting mitochondrial fragmentation, whereas AB_1372 showed only a slightly increased depolarization rate of 4.6% (Fig. 2F).

Genome comparison revealed genome sizes of 3.84 Mbp for AB_1372 and 3.92 Mbp for AB_2778, with 3,695 and 3,763 coding genes, respectively (Fig. S4). For 3,425 of these genes a homologue was found in both genomes; among those were 75 virulence genes. Although the two isolates belong to the same IC, more than 8,000 SNVs were detected, indicating both genetic diversity within the IC and that the different virulence phenotypes cannot be attributed to differences in specific genes.

Taken together, the results show that while biofilm formation, growth, and adhesion rates were similar in both isolates, AB_2778 showed a significantly higher potential in mediating apoptosis and targeting mitochondria.

### Complexome profiling of mitochondrial protein complexes upon infection with A. baumannii.

Mitochondria are important organelles containing metabolic pathways required for the energy supply of the cell and the production of central metabolites for anabolic pathways and cofactors. Upon stress, mitochondria can adapt via mitogenesis, regulation of the respiratory chain, or changes in mitochondrial morphology and dynamics. In severe stress situations, mitochondria can induce intrinsic apoptosis by increasing mitochondrial permeability, releasing cytochrome *c*, and activating caspase-9 (24–26).

As mitochondria might be a relevant target during infection with A. baumannii, complexome profiling of enriched mitochondrial fractions of HUVECs was employed. This method allows the identification of stable protein assemblies and subassemblies and the investigation of the mitochondrial response to stress upon infection. Enriched mitochondrial fractions of HUVECs infected with AB_2778 and AB_1372 and uninfected HUVECs were used. The enriched mitochondrial fractions were treated with mild solubilization buffers, followed by separation of mitochondrial complexes by blue native gel electrophoresis. This procedure isolates stable mitochondrial complexes, supercomplexes, and assembly intermediates according to their molecular mass (Fig. S5). Native gels were sliced into 48 fractions from high to low molecular weights covering a mass range of approximately 10 to 10,000 kDa and proceeded to mass spectrometry to identify and quantify proteins. Intensity-based absolute quantification (IBAQ) values were recorded and served as a measure of total protein abundance. The combined data table of all samples includes 4,431 identified proteins, 721 from A. baumannii and 3,710 human; 911 proteins were mitochondrial (according to the Gene Ontology cellular component database). Quantifying proteins from cellular compartments in each sample showed fewer mitochondrial proteins in HUVECs infected by the virulent strain (Fig. S6), whereas the endoplasmic reticulum and the Golgi apparatus were not severely affected upon infection with both isolates (Fig. S7). This suggests that infection of HUVECs with AB_2778 leads to the disintegration of mitochondria.

### Reduction of OXPHOS complexes in mitochondria upon infection with highly virulent A. baumannii AB_2778.

**FIG 3:**
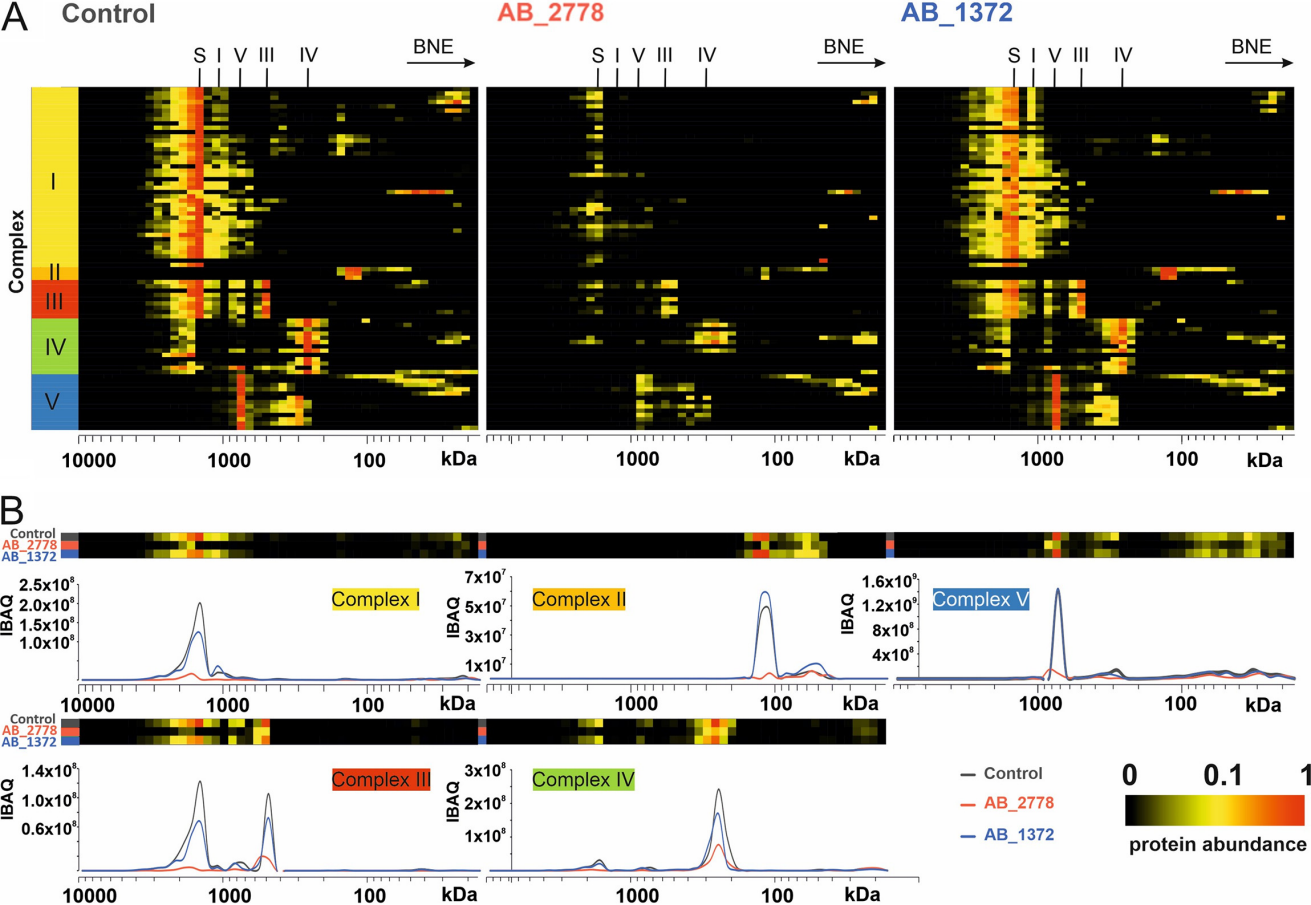
Stability of OXPHOS complexes of HUVECs upon infection with a virulent (AB_2778) or nonvirulent (AB_1372) strain of A. baumannii compared to that of uninfected cells (control). (A) Migration profiles of OXPHOS complexes of uninfected HUVECs compared to AB_1372- and AB_2778-infected HUVECs. (B) Alignment of protein migration profiles of each OXPHOS complex obtained from uninfected HUVECs and after infection with AB_1372 and AB_2778. Two-dimensional (2D) plots indicate the sum of all intensity-based absolute quantification (IBAQ) values for each OXPHOS complex. BNE, blue native electrophoresis; S, supercomplex; I to V, numbers of complexes of the respiratory chain.

In intact mitochondria, the OXPHOS machinery is responsible for generating the MMP and ATP synthesis. It consists of four complexes (complexes I to IV [CI to CIV]) located at the inner mitochondrial membrane building the electron transport chain (ETC) and the ATP synthase (complex V [CV]). The ETC generates a proton gradient between the mitochondrial matrix and the mitochondrial intermembrane space, which the ATP synthase uses during oxidative phosphorylation (OXPHOS) to generate ATP. All complexes involved in OXPHOS were detected in the complexome profiling and were stable in uninfected HUVECs (Fig. 3A, control).

Loss of MMP leads to an interruption of ATP production. Further disintegration of mitochondrial structure may lead to a release of cytochrome *c* from the intermembrane space and inner crista compartment into the cytosol, followed by the formation of the apoptosome (protein complex that binds and activates procaspase-9). A heat map of normalized IBAQ values of the OXPHOS complexes exhibited a decrease in respiratory chain complexes, supercomplexes, and ATP synthase upon infection with the virulent isolate AB_2778 (Fig. 3A). The control cells and, to a lesser extent, AB_1372-infected cells showed the typical assembly of CI intermediates, indicating active CI biosynthesis. Upon infection with AB_2778, no building blocks of CI could be detected, suggesting stalled *de novo* complex formation. This was also shown by the total abundance values of subunits (Fig. 3B). The total amount (the sum of all IBAQ values of all subunits) was reduced to 10.3% for CI, 16.6% for CII, 12.8% for CIII, 34.6% for CIV, and 30.8% for CV compared to the uninfected control. The amount of OXPHOS complexes in HUVECs infected with the least virulent isolate, AB_1372, was minimally reduced, with comparable patterns of single complexes and supercomplexes (Fig. 3A, AB_1372). Total abundances were reduced to 76.9% for CI, 67.0% for CIII, 71.2% for CIV, and 91.4% for CV. CII subunits were the only proteins showing an increase (136.9%) upon infection with AB_1372, suggesting a compensatory response to mild mitochondrial targeting. The abundances of all complexes of the ETC were reduced to 26.7% (AB_2778) and 86.2% (AB_1372), indicating a reduction of the complexes in an isolate-dependent manner. As both stable MMP and energy supply are crucial for cells to survive, controlling the cellular mitochondrial protein synthesis seems to be an important determinant of the pathogenicity of A. baumannii.

### Infection with highly virulent A. baumannii AB_2778 disturbs biogenesis of OXPHOS complexes.

The biosynthesis and assembly of mitochondrial membrane proteins are multifaceted and tightly controlled. Additional assembly factors, chaperones, and an intact import machinery are crucial to ensure the correct insertion of these polytropic proteins with their prosthetic groups leading to functional complexes (27, 28). The subunits of the OXPHOS complexes are encoded in both the nuclear (nDNA) and mitochondrial (mtDNA) genome, except for CII (encoded entirely by nDNA). CI contains seven subunits, CIII one subunit, CIV three subunits, and CV two subunits encoded by mtDNA (29).

**FIG 4:**
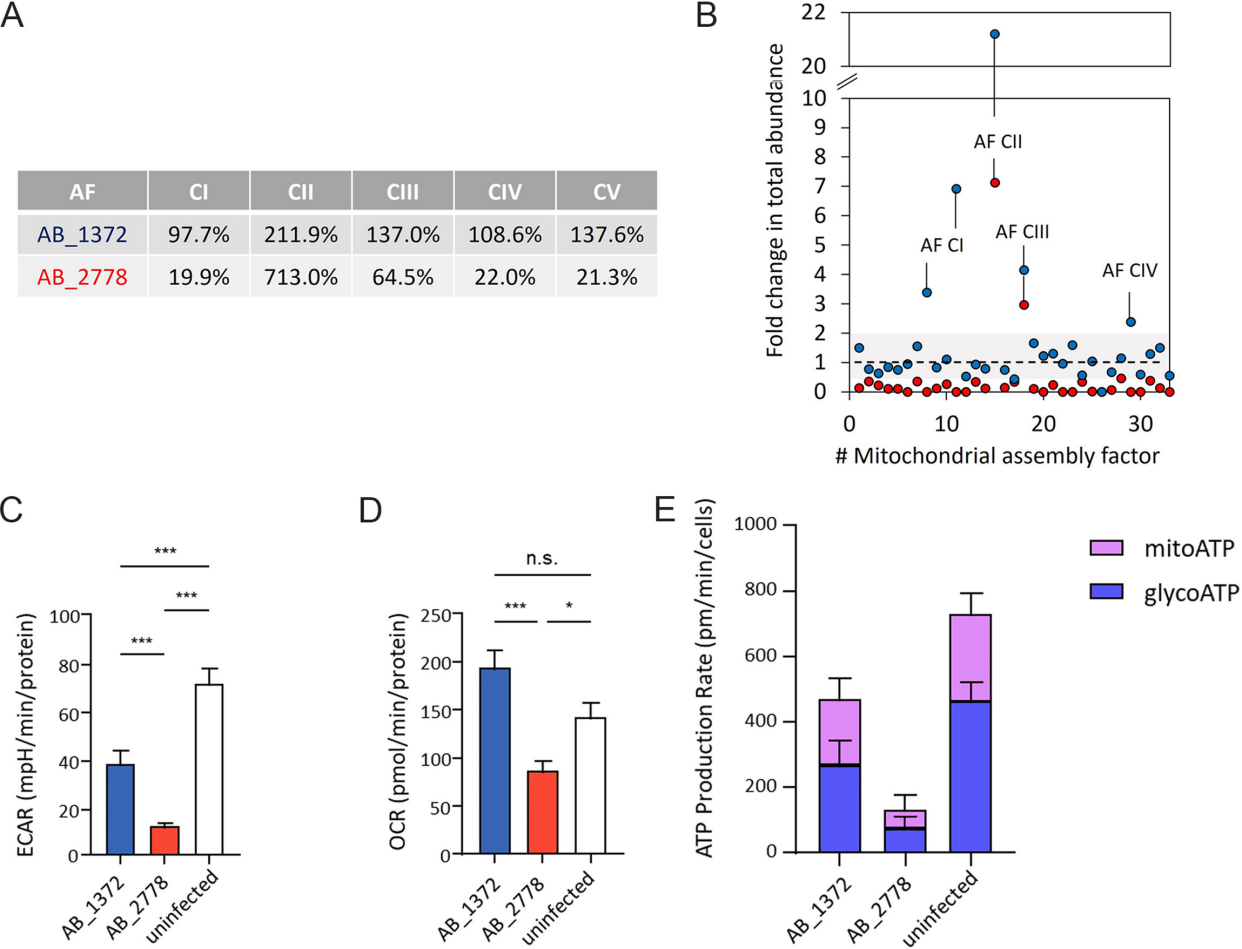
Regulation of mitochondrial assembly factors. (A) Values display the ratio of total abundance of the assembly factors (AF) of complexes I to V (CI to CV) after infection with AB_2778 and AB_1372 and the control. (B) Each dot represents an assembly factor of the OXPHOS. Fold changes in total abundance indicate the sum of all IBAQ values for one AF of the infected HUVECs divided by the sum of IBAQ values of uninfected HUVECs. Blue dots, AB_1372; red dots, AB_2778. (C to E) High-resolution respirometry of HUVECs infected with AB_1372 or AB_2778 or left uninfected. Shown are determinations of the extracellular acidification rate (ECAR), oxygen consumption rate (OCR), and ATP production rate. Values are means ± SD (*n* = 3). Statistical analysis was performed by a two-tailed *t* test. *, *P < *0.05; **, *P < *0.01; ***, *P < *0.005.

Infection with the virulent A. baumannii isolate AB_2778 reduced the total abundance of mitochondrial assembly factors (Fig. 4A). Assembly factors required for biogenesis of CI were reduced to 19.9%. Assembly factors were down to 64.5% for CIII, 22.0% for CIV, and 21.3% for CV. Notably, SDHAF2, an assembly factor of CII, increased 7.1-fold upon infection with AB_2778. In contrast, AB_1372 assembly factors increased compared to the control or showed a minor reduction or no change (Fig. 4B). However, SDHAF2 increased the most (2.1-fold) compared to the control upon infection with AB_1372. For CI, assembly factors showed no change (97.7%), whereas for CIII to CV, assembly factors increased (CIII, 137.0%; CIV, 108.6%; and CV, 137.6%). The decrease of assembly factors and OXPHOS complexes upon infection with AB_2778 and AB_1372, respectively, indicates not only a reduction of the complexes themselves but also a disturbance of the biogenesis of the complexes in an isolate-dependent manner.

### Metabolic adaptations of human host cells upon infection with A. baumannii.

Complexome profiling indicated the reduction of mitochondria in human host cells upon infection with AB_2778. This raised the question of whether the host cell metabolism is reprogrammed by highly virulent A. baumannii. High-resolution respirometry was performed to quantify the metabolic adaptations upon infection. For this purpose, the basal oxygen consumption rate (OCR), extracellular acidification rate (ECAR), and ATP production rate were measured. OCR measurements allow direct quantification of mitochondrial respiration and ECAR the quantification of glycolysis.

Infection with A. baumannii led to reduced ECAR in HUVECs, indicating reduced glycolysis (Fig. 4C). AB_1372 reduced ECAR significantly, to 38.0 milli potential of hydrogen (mph)/min/protein, compared to that for the uninfected control (71.4 mpH/min/protein), whereas AB_2778 further decreased the level of ECAR to 12.1 ± 2.8 mpH/min/protein. OCR measurements showed that AB_1372 increased mitochondrial respiration in HUVECs slightly compared to that for the uninfected control (Fig. 4D). In contrast, AB_2778 decreased the metabolic activity significantly, to 6.8 pmol/min/protein, which is in line with the reduction of ETC complexes detected in the complexome profiling (Fig. 3). A. baumannii-infected HUVECs exhibited reduced ATP production rates compared to those of the uninfected control (Fig. 4E), which was more pronounced in the case of AB_2778. The addition of inhibitors during measurement enabled the discrimination of ATP produced via glycolysis (glycoATP) and via the respiratory chain (mitoATP) to evaluate the functionality of the respiratory chain complexes. Infection with both A. baumannii isolates reduced glycoATP production, reflecting the decreased ECAR. In contrast, after infection with AB_1372, mitoATP rates were less severely affected, which is in line with the significantly decreased ECAR and relatively unaltered OCR of AB_1372.

In summary, metabolic activity was decreased, with lower glycolytic rates and mitoATP production, in human host cells upon infection with both A. baumannii isolates. The substantial reduction of OCR was exclusive to the highly virulent strain AB_2778 and likely accounts for its severely decreased mitoATP production rate.

### Infection with highly virulent A. baumannii leads to reduced protein biosynthesis in mitochondria.

**FIG 5:**
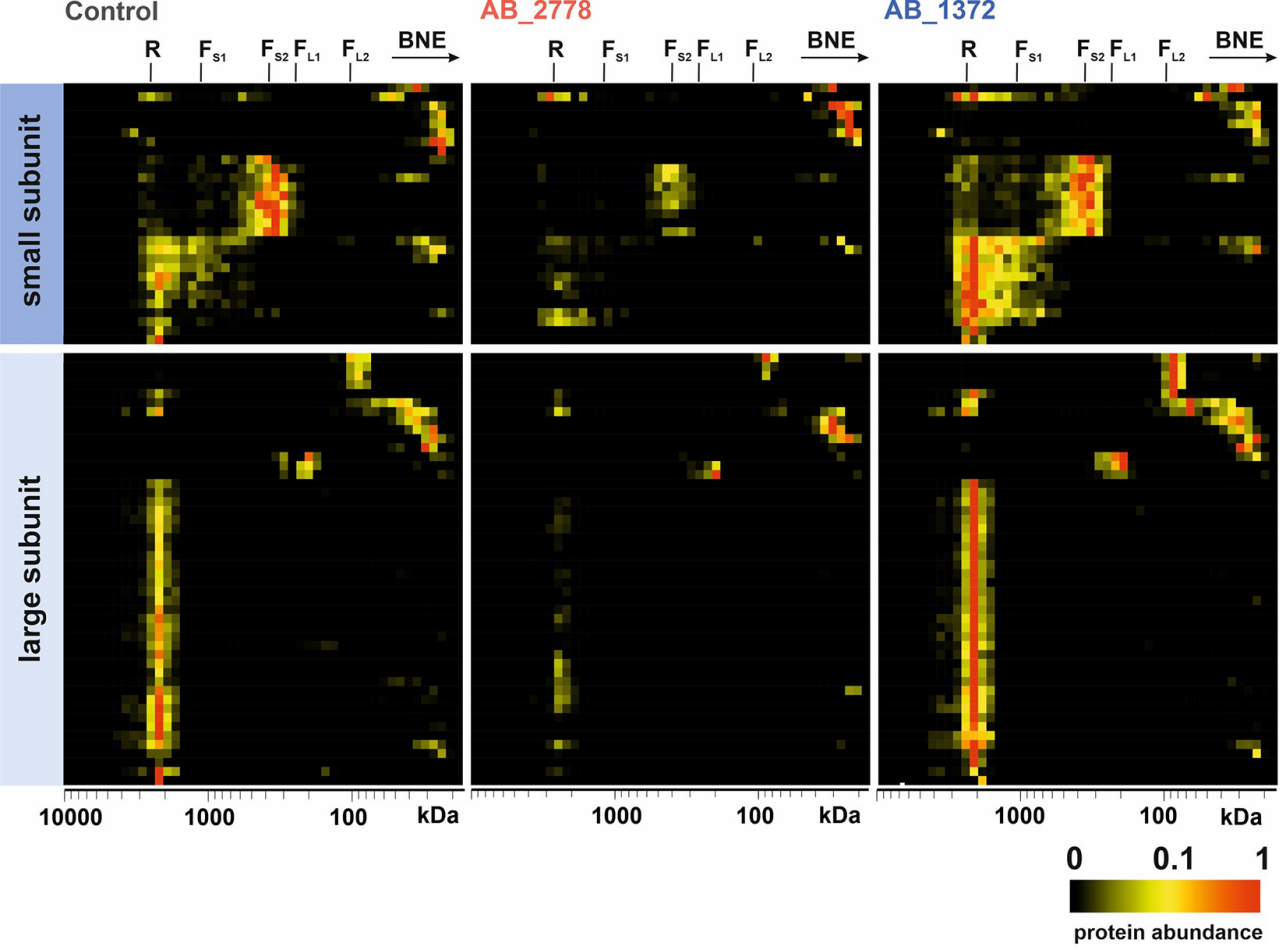
Stability of mitochondrial ribosomes of HUVECs upon infection with AB_2778 and AB_1372 compared to that of uninfected HUVECs (control). The heat map of protein abundance distribution of the small and large subunits of ribosomes in blue native gel electrophoresis exhibits a decrease of ribosomal complexes upon infection with AB_2778 compared to AB_1372 and the uninfected control. R, ribosomes; F_S1_, small fragment 1; F_S2_, small fragment 2; F_L1_, large fragment 1; F_L2_, large fragment 2.

Mitochondrial ribosomes (mitoribosomes) are required to translate proteins encoded in the mtDNA. These proteins are part of the ETC and involved in ATP production. Analysis of mitoribosomes indicated that infection of HUVECs with AB_2778 led to a drop in mitoribosomal complexes, suggesting that compensation of dysfunctional OXPHOS via an increased mitochondrial biosynthesis was not functioning (Fig. 5, AB_2778). Total abundances decreased to 46.2% for the small subunit and 30.6% for the large subunit compared to those of the uninfected control. The sum of total abundances of all subunits of mitoribosomes was down to 35.9%. It seemed that AB_1372 had less impact on mitoribosomal complexes. Total abundances increased to 137.8% for the small subunit and 148.3% for the large subunit. In total, mitoribosomes increased to 144.8% compared to those of uninfected HUVECs, assuming a response and compensation by increased biosynthesis of mitochondrial proteins (Fig. 5, AB_1372).

### A. baumannii influences mitochondrial morphology and mitophagy.

**FIG 6:**
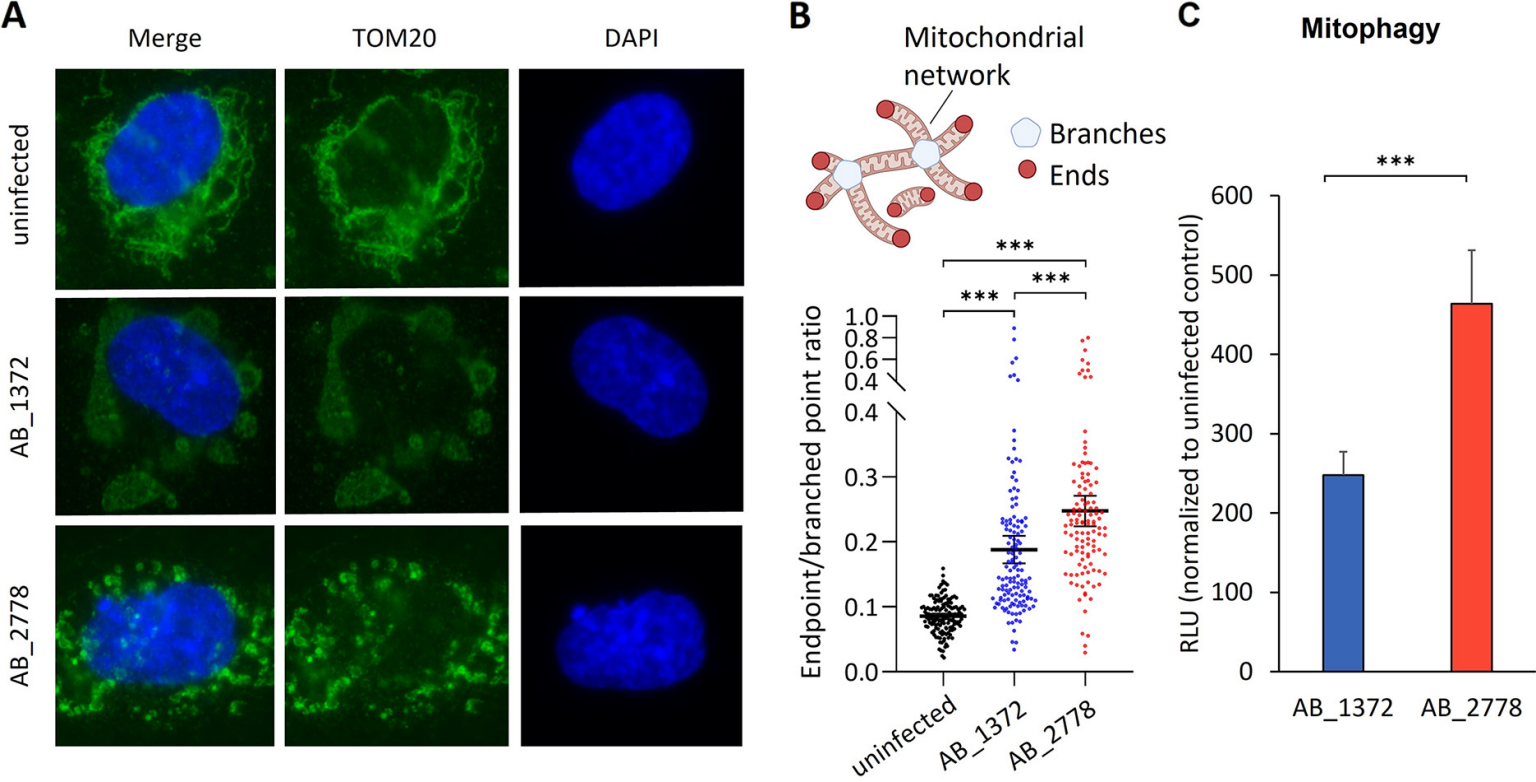
Changes in morphology and metabolic adaptation of mitochondria upon infection with AB_1372 and AB_2778. (A) Representative image of fluorescence microscopy of uninfected and infected HUVECs. DAPI staining was used for visualization of DNA. The outer mitochondrial membrane protein TOM20 was stained in an immunofluorescence assay, and mitochondrial morphology and dynamics were monitored. (B) Quantification of the mitochondrial endpoint/branched point ratio (EBR) in HUVECs infected with A. baumannii AB_1372 or AB_2778 for 16 h in comparison to uninfected cells. EBR is defined as the sum of all endpoints divided by the sum of all branched points (32, 33). Data are presented as means with 95% confidence intervals from approximately 150 cells quantified for each condition (*n* = 3). Statistical analyses were performed using a Kruskal-Wallis test with Dunn’s multiple-comparison test. Values are means ± 95% confidence intervals. (C) Mitophagy was determined by fluorometry using the Dojindo mitophagy detection kit. Values are means ± SEM (*n* = 3). Statistical analysis was performed by a two-tailed *t* test. ***, *P* < 0.005.

Complexome profiling of A. baumannii-infected HUVECs revealed mitochondria as a main target. To evaluate morphological changes upon infection, the outer mitochondrial membrane protein TOM20 was immunostained (30). For the uninfected control, a high interconnection of a tubular mitochondrial network was observed, which indicates balanced fusion and fission (Fig. 6A, first row). Infection with AB_1372 led to a morphological change of mitochondria from filamentous to bleb-like structures (Fig. 6A, second row, and Fig. S8A). In contrast, infection with AB_2778 led to perturbation of the mitochondrial network and resulted in smaller and condensed structures of mitochondria (Fig. 6A, third row, and Fig. S8A).

Mitochondrial fission is predominantly mediated by the dynamin-related protein 1 (DRP-1), whereas mitochondrial fusion is controlled by optic atrophy protein 1 (OPA1) (31). Analysis of the complexome data revealed a higher abundance of DRP-1 upon infection with AB_2778 (Fig. S9). Blocking of DRP-1 by the chemical inhibitor Mdivi-1 prevented the disruption of mitochondrial network in AB_2778-infected HUVECs, indicating DRP-1-dependent fragmentation (Fig. S8B). In contrast, AB_1372-infected cells showed a higher abundance of OPA1, indicating mitochondrial fusion, which could not be prevented by Mdivi-1 treatment.

To further assess the impact on mitochondrial morphology on infected HUVECs, the length and branching of mitochondrial networks were analyzed from TOM20 immunostainings and the endpoint/branched point ratio (EBR) was calculated (32, 33) (Fig. 6B). In uninfected cells, the EBR was low, reflective of few endpoints and extensive branching. In contrast, the number of endpoints increased upon infection with AB_1372 and significantly more with AB_2778, indicating reduced network connectivity and more extensive fragmentation (Fig. 6B). The outer membrane protein OmpA of A. baumannii is an important virulence factor and causes mitochondrial fragmentation (10). However, no significant differences in relative *ompA* expression levels could be detected between AB_2778 and AB_1372 (Fig. S10).

Fragmentation of mitochondria is followed by decomposition of impaired mitochondria during mitophagy (34). Fission of mitochondria in AB_2778-infected HUVECs promotes mitophagy significantly (463.5 relative luminescence units [RLU]) compared to that for AB_1372-infected cells (247.5 RLU). This suggests the removal of damaged mitochondria upon infection, while fusion of mitochondria might help to maintain its function under stress conditions in the case of AB_1372.

## DISCUSSION

We analyzed virulence traits of A. baumannii clinical isolates belonging to the most widely disseminated international clonal lineage, IC2 (1). *In vitro* and *in vivo* data identified AB_2778 as highly virulent and AB_1372 as the least virulent isolate. For our study, we selected patient isolates which exhibit all characteristics of the worldwide most prevalent and widely disseminated clonal lineage IC2. The widely used reference strain ATCC 19606^T^ differs from these clinical isolates since it (i) exhibits no XDR phenotype, (ii) does not belong to a major clonal lineage, and (iii) has low pathogenicity (35). Most studies investigating virulence of A. baumannii have been performed with reference strains and are likely not applicable to clinical isolates. Furthermore, many studies do not compare different clinical isolates of A. baumannii. Comparison of the least virulent isolate to a highly virulent isolate enabled us to identify different interactions of A. baumannii with HUVECs indicated by morphological and metabolic changes of mitochondria.

The extensive change in morphology and severe restriction of functionality show that mitochondria are the main target organelle of clinical A. baumannii. Two possible mechanisms can be envisioned: the interaction of intracellular bacteria directly mediates this phenotype and/or secreted outer membrane vesicles (OMVs) play a crucial role. This is in line with previous studies demonstrating that OmpA is important to induce mitochondrial fragmentation and that not only whole bacterial cells but also OMVs containing OmpA are sufficient for this mechanism (10, 36, 37). While we did not detect significant differences in *ompA* expression between AB_1372 and AB_2778, further studies should investigate the role of this important pathogenicity factor during mitochondrial remodeling and to what extent clinical isolates differ from references strains. In particular, regulation of *ompA* expression should be further investigated, as previous data indicate reduced gene expression in a murine infection model (38). Not only A. baumannii has evolved strategies to perturb this organelle to modulate host cell response: OMVs of Neisseria gonorrhoeae target mitochondria and induce apoptosis (37), and those of Chlamydia trachomatis modify mitochondrial morphology but also the endoplasmic reticulum and the Golgi apparatus (39, 40).

Further studies should determine which factors of the highly virulent AB_2778 target mitochondria and to what extent they differ from the least virulent strain, AB_1372. However, this might prove difficult since this could be due to a complex expression phenotype or posttranslational modifications of many proteins. Furthermore, A. baumannii strains have plastic virulence phenotypes since they can spontaneously switch from low to high pathogenicities (41). The number of virulence genes within the genome of the clinical strains is clearly not decisive and did not correlate with the phenotype. This was also shown previously for carbapenem-resistant clinical isolates of Escherichia coli and Klebsiella pneumoniae *in vitro* and *in vivo* (42).

A study comparing a virulent, type IV secretion system (T4SS)-positive strain of Legionella pneumophila to an avirulent T4SS-negative isolate described the induction of glycolysis in macrophages after infection. T4SS-dependent mitochondrial fission and fragmentation were reported with subsequent impairment of OXPHOS and induction of a Warburg-like metabolism within the host cell (43). Here, we have described the reduction of glycolysis upon A. baumannii infection with an isolate-specific impact on the functionality of ETC complexes. Complexome profiling demonstrated that not only existing mitochondria but also the assembly of the complexes of the ETC are targets of A. baumannii infection. The detection of increased SDHAF2 indicates the biogenesis of functional CII of the respiratory chain upon infection with A. baumannii AB_1372 (Fig. 4B). Increased IBAQ values confirmed the high abundance of CII. Compared to other OXPHOS complexes, CII has specific characteristics. It is the only OXPHOS complex without proton pumping activity (44) and further connects the tricarboxylic acid cycle (TCA) and ETC by introducing electrons resulting from the conversion of succinate to fumarate. Infection with AB_1372 decreased ECAR, which might indicate reduction of the TCA (Fig. 6). In line with the values for ECAR, glycoATP rates were more reduced than mitoATP rates, leading to the hypothesis that increased amounts of CII could convert the remaining succinate to fumarate, thereby providing electrons for the ETC and maintaining the production of mitoATP.

The reduced levels of mitochondrial complexes and the lower mitochondrial function indicate a reduction of the mitochondrial mass. It seems that the elimination of mitochondria by macrodegeneration processes like mitophagy is triggered by AB_2778 (Fig. 6). Controlled clearance by mitophagy is an important process to avoid accumulation of nonfunctional mitochondria. To date, we cannot distinguish if a mitochondrial dysfunction leads to increased elimination of mitochondria, the fragmentation of the mitochondrial network, an elevation of mitophagy due to infection-dependent signals, or combinations thereof.

Generally, it is assumed that A. baumannii is an extracellular bacterium (45–47). However, very recently, the incidence of an intracellular niche of highly invasive A. baumannii was described; this niche allowed replication of the bacteria and could enable a direct interaction of bacteria with mitochondria (48). It should be determined if the impaired OXPHOS upon infection provokes a Warburg-like effect leading to the production of lactate, which could serve as a carbon source for A. baumannii and enhance the intracellular replication of the bacteria.

Our study is the first report of complexome profiling of A. baumannii-infected endothelial cells revealing an isolate-dependent impact on biogenesis, functionality, and selective degradation of mitochondria with subsequent metabolic adaptation of the host cells.

## MATERIALS AND METHODS

### Bacterial isolates and antimicrobial susceptibility testing.

Clinical A. baumannii isolates were recovered from patients hospitalized at the Goethe University Hospital in Frankfurt, Germany. Inclusion criteria were the presence of OXA-23 with phenotypic imipenem resistance of A. baumannii belonging to clonal lineage IC2. A. baumannii reference strain AB_5075 was purchased from the Manoil lab (University of Washington, Seattle, WA) and ATCC 19606^T^ from the DSMZ (German Collection of Microorganisms and Cell Culture, Braunschweig, Germany). Antimicrobial susceptibility was determined by antibiotic gradient strips (Liofilchem, Roseto degli Abruzzi, Italy, or bioMérieux, Nürtingen, Germany) and broth microdilution in case of colistin using cation-adjusted Mueller-Hinton broth. MICs were evaluated and interpreted according to EUCAST (https://www.eucast.org/clinical_breakpoints/) and CLSI (49) guidelines.

### Genome sequencing and computational analysis.

Genome sequencing was carried out for all isolates using short-read technology (MiSeq platform; Illumina, San Diego, CA) as previously described (50, 51). For isolates AB_1372 and AB_2778, long-read technology (MinION platform, Oxford Nanopore Technologies, Oxford, UK) was applied to generated hybrid assemblies (see the supplemental material). The genome sequences of all isolates were deposited publicly in the NCBI database (BioProject number PRJNA901493). The obtained assemblies were annotated using Prokka version 1.14.6 (52), and multilocus sequence types were determined using the software mlst version 2.18.0 (https://github.com/tseemann/mlst) utilizing typing schemes from the PubMLST database (https://pubmlst.org/). Screening of assemblies for antibiotic resistance genes and virulence genes was carried out using ABRicate version 1.0.1 (https://github.com/tseemann/abricate). For antimicrobial resistance genes, NCBI ResFinder (https://cge.food.dtu.dk/services/ResFinder/) was used, applying thresholds of ≥80% gene coverage and ≥80% nucleotide sequence identity. For virulence genes, a custom database was constructed based on all entries for A. baumannii in PATRIC 3.6.12 (https://www.patricbrc.org; accessed 4 April 2022) and an in-house list including additional 95 genes. Thresholds of 90% for both coverage and nucleotide sequence identity were applied. Pan-genome analysis was carried out using Roary version 3.13.0 (53). Nucleotide alignments of the core genome were used to construct a maximum likelihood tree with fasttree version 2.1.10 using a general time reversible model (54). To access support of the nodes, 100 random bootstrap replicates were performed.

### Analysis of virulence traits of A. baumannii.

Evaluation of growth kinetics, quantification of bacterial biofilm using the crystal violet method, assessment of bacterial adhesion to HUVECs, and detection of caspase activation in HUVECs upon infection are described in the supplemental material.

### Cultivation and infection of human endothelial cells.

Primary human umbilical cord vein cells (HUVECs) from healthy donors were cultivated in collagenized 75-cm^2^ cell culture flasks using endothelial cell growth medium with supplement mix (ECGM; PromoCell, Heidelberg, Germany) and 10% fetal calf serum (FCS; Sigma-Aldrich, Darmstadt, Germany). Cell culture flasks were incubated in a 5% CO_2_ atmosphere at 37°C and a humidity of 95%. For infection experiments, HUVECs were seeded into collagenized 6-well plates or 96-well plates at a density of 5 × 10^5^ or 1 × 10^4^ cells per well, respectively. Bacterial cultures were adjusted to an optical density at 600 nm (OD_600_) of 0.05 in lysogeny broth (LB) and incubated at 37°C until an OD_600_ of 0.2 was reached. Thereafter, bacteria were centrifuged at 3,000 × *g* for 20 min and washed with phosphate-buffered saline (PBS). Bacteria were resuspended in ECGM and adjusted to the required multiplicity of infection (MOI). After infection of HUVECs, cell culture plates were centrifuged for 5 min at 300 × *g* and incubated for defined time points.

### Analysis of cell death using annexin V/PI.

HUVECs were seeded into 6-well plates and infected with A. baumannii (MOI, 100) or incubated with 1 μM camptothecin as a positive control. Thereafter, plates were incubated for 4, 8, and 16 h (37°C and 5% CO_2_) and the supernatant was centrifuged (3,000 × *g* for 10 s). Adherent cells were washed with PBS and detached from the wells using 0.05% trypsin-EDTA. Sediments of the centrifuged supernatant were mixed with the detached cells and washed twice. After centrifugation (3,000 × *g* for 10 s) and resuspension in annexin binding buffer, cells were stained with annexin V-fluorescein isothiocyanate (FITC) and propidium iodide (BD Biosciences, Heidelberg, Germany). Induction of apoptosis and necrosis was monitored by flow cytometry (BD FACSVerse).

### Analysis of mitochondrial membrane depolarization using DilC1(5).

HUVECs were infected exactly as described for the annexin V/PI assay. Thereafter, HUVECs were stained with 10 μM DilC1(5) (immunostep) and incubated for 15 min (37°C and 5% CO_2_). After incubation, induction of mitochondrial membrane depolarization was monitored by flow cytometry (BD FACSVerse).

### Evaluation of bacterial virulence using the Galleria mellonella *in vivo* infection model.

Larvae of the greater wax moth (Galleria mellonella) were injected with 10 μL of bacterial suspensions corresponding to 10^3^ to 10^7^ CFU via the last left proleg using a Hamilton precision syringe. Infected larvae were incubated at 37°C and survival was monitored for 72 h. Time-kill curves were generated and the median lethal dose (LD_50_) was calculated after 24 h by nonlinear regression analysis as described previously (55). For details, see the supplemental material.

### Immunostaining and morphological analysis of infected HUVECs.

HUVECs were seeded on coverslips and thereafter infected with A. baumannii for 16 h (MOI, 100) in ECGM. The supernatant was discarded and coverslips were incubated with 3.75% paraformaldehyde for fixation. Slides were washed with PBS and blocked with 1% bovine serum albumin (BSA) in PBS for 1 h at room temperature (RT). Cells were washed and incubated with 0.2% Triton X-100 in PBS for 15 min at RT. Samples were stained with primary antibody (1:200 in PBS; anti-TOM20 antibody ab56783, anti-GM130 antibody 59890S, or anti-calnexin antibody 23198S; Abcam) overnight at 4°C and with the secondary anti-mouse Alexa Fluor 488 antibody (1:200 in PBS; Agilent Dako) for 1 h at RT. Subsequently, DNA was stained with 4′,6-diamidino-2-phenylindole (DAPI) and the actin cytoskeleton with tetramethylrhodamine (TRITC)-phalloidin. Samples were mounted on glass slides using Fluoprep (Agilent Dako) and analyzed by fluorescence microscopy (Nikon Eclipse Ci-L). The branching status of mitochondrial network was determined by calculating the mean of endpoint/branched point ratio (EBR) of mitochondrial particles in approximately 150 cells for each condition, employing a software pipeline as previously described (32, 33, 56). For analysis of mitophagy, HUVECs were seeded into 96-well plates and infected with A. baumannii (MOI, 100) in ECGM. Mitophagy was determined by fluorometry using mitophagy detection kit MD01 (Dojindo, Munich, Germany) according to the manufacturer’s instructions.

### Measurement of cellular metabolism/ATP rate measurement.

HUVECs were seeded overnight at 1 × 10^5^ cells per well in 50 μL of ECGM with 10% fetal calf serum (FCS) on XF96 PDL cell culture microplates (Agilent Technologies). Subsequently, HUVECs were infected with bacteria (MOI, 100) and incubated for 16 h. The supernatant was discarded and HUVECs were washed with medium and treated with 10 mg/L of colistin sulfate (in ECGM) for 30 min to ensure that there was no interference with bacterial metabolism during measurement. Afterwards, culture medium was replaced with Seahorse XF Dulbecco modified Eagle medium (DMEM) supplemented with 10 mM glucose and 2 mM glutamine (all from Agilent Technologies). The plate was equilibrated for 30 min in a non-CO_2_ incubator at 37°C. Oxygen consumption and extracellular acidification were measured on a Seahorse XFe 96 extracellular flux analyzer (Agilent Technologies). Five cycles were performed per injection, each consisting of 3 min of measurement and 30 s of mixing. To analyze the ATP production rate, 2.5 μM oligomycin and 500 nM rotenone together with antimycin A were sequentially added to the cells (all from Cayman Chemical, Ann Arbor, MI). Data were normalized to protein and processed using Wave Desktop (version 2.6.0.31). ATP rates were calculated using the Seahorse Analytics online tool (version from March 2022). Presented data were composed from three independent experiments.

### Complexome profiling.

Mass spectrometry raw data, identification and quantification of proteins, and native mass calibration together with a detailed description of the procedure have been deposited to the ProteomeXchange Consortium via PRoteomics IDEntification Database (PRIDE) repository (https://www.ebi.ac.uk/pride/archive) (57) with the data set identifier PXD035235. Data were loaded to Perseus proteomics software (58) and gene ontology terms for cellular components were added to each protein. The sum of IBAQ values was used to quantify cellular compartments and generating pie charts.

### Ethics statement.

All bacterial strains were isolated as part of routine microbiological diagnostics and stored in an anonymized database. According to the Ethics Committee of the Hospital of Johann Wolfgang Goethe-University, Frankfurt am Main, no informed consent or ethical approval of the study was necessary. Local data protection rules were strictly followed (§12 Hessisches Krankenhausgesetz and §33 Hessischer Datenschutzgesetz).

### Data availability.

Mass spectrometry raw data, including a detailed description of the procedure, have been deposited in the ProteomeXchange Consortium via PRoteomics IDEntification Database (PRIDE) repository (https://www.ebi.ac.uk/pride/archive) with the data set identifier PXD035235. Genome sequencing data generated in this study were deposited in the NCBI Sequence Read Archive (SRA), accessible under BioProject number PRJNA901493.
