# Supplementary material for: Infection of Endothelial Cells with Acinetobacter baumannii Reveals Remodelling of Mitochondrial Protein Complexes
Source: Microbiol Spectr. 2023 Apr 13;11(3):e05174-22. doi: 10.1128/spectrum.05174-22 (PMC10269660; doi:10.1128/spectrum.05174-22)
Supplement: Supplemental file 1 — Fig. S1 to S10, Table S1, and supplemental methods. Download spectrum.05174-22-s0001.pdf, PDF file, 1.6 MB [file spectrum.05174-22-s0001.pdf]

Supplemental Material

Infection of endothelial cells with *Acinetobacter baumannii* reveals remodelling of mitochondrial protein complexes

Laura Leukert[1], Manuela Tietgen[1,2], Felix F. Krause[1,3], Tilman G. Schultze[1], Dominik C. Fuhrmann[4], Charline Debruyne[5], Suzana P. Salcedo[5], Alexander Visekruna[3], Ilka Wittig[6], Stephan Göttig[1,#]

[1]Institute for Medical Microbiology and Infection Control, University Hospital, Goethe University, Frankfurt am Main, Germany
[2]University Center of Competence for Infection Control of the State of Hesse, Frankfurt am Main, Germany
[3]Institute for Medical Microbiology and Hygiene, Philipps-University, Marburg, Germany
[4]Institute of Biochemistry I, Faculty of Medicine, Goethe University, Frankfurt am Main, Germany
[5]Laboratory of Molecular Microbiology and Structural Biochemistry, Centre National de la Recherche Scientifique UMR5086, Université de Lyon, Lyon, France
[6]Functional Proteomics, Institute of Cardiovascular Physiology, Goethe University, Frankfurt am Main, Germany

#Corresponding author: stephan.goettig@kgu.de

Supplementary Figures

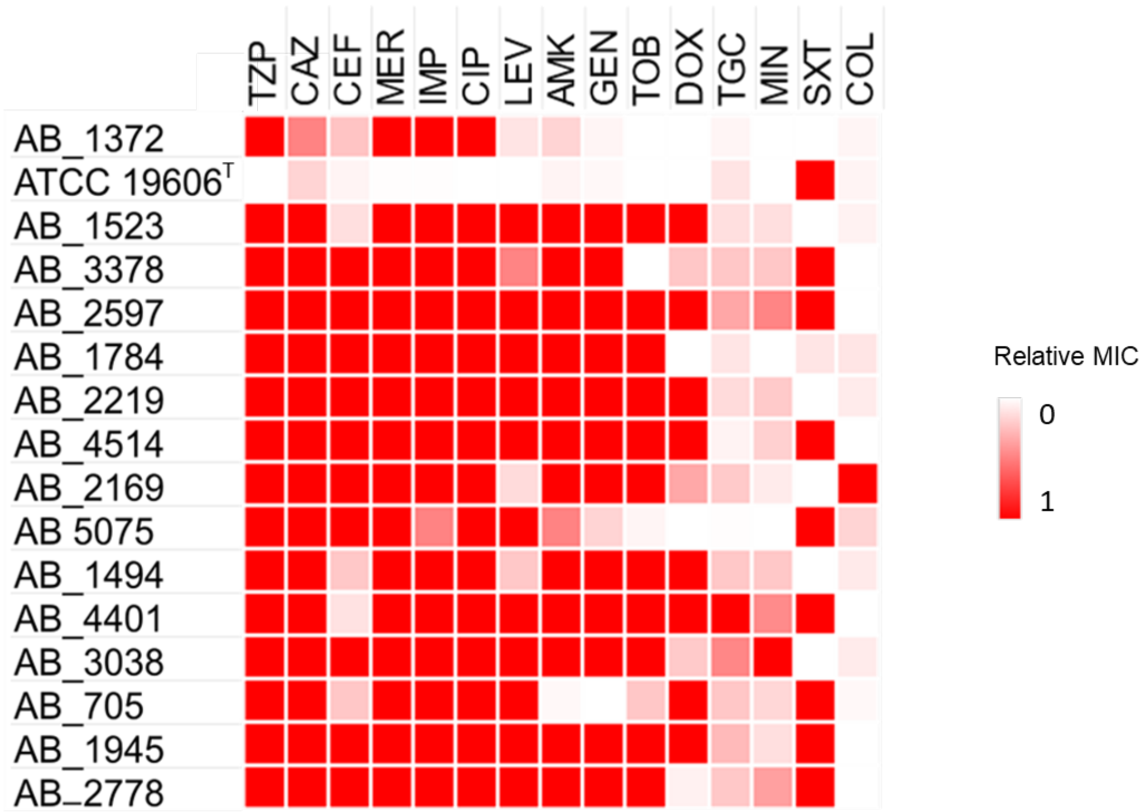

FIG S1 Antimicrobial susceptibility profile. The relative minimum inhibitory concentration (MIC) indicates the ratio of the actual MIC of an isolate divided by the highest MIC value for the respective antibiotic in the data set. The color bar displays the values. TZP, piperacillin/tazobactam; CAZ, ceftazidime; CEF, cefepime; MER, meropenem; IMP, imipenem; CIP, ciprofloxacin; LEV, levofloxacin; AMK, amikacin; GEN, gentamicin; TOB, tobramycin; DOX, doxycycline; TGC, tigecycline; MIN, minocycline; SXT, trimethoprim-sulfamethoxazole; COL, Colistin.

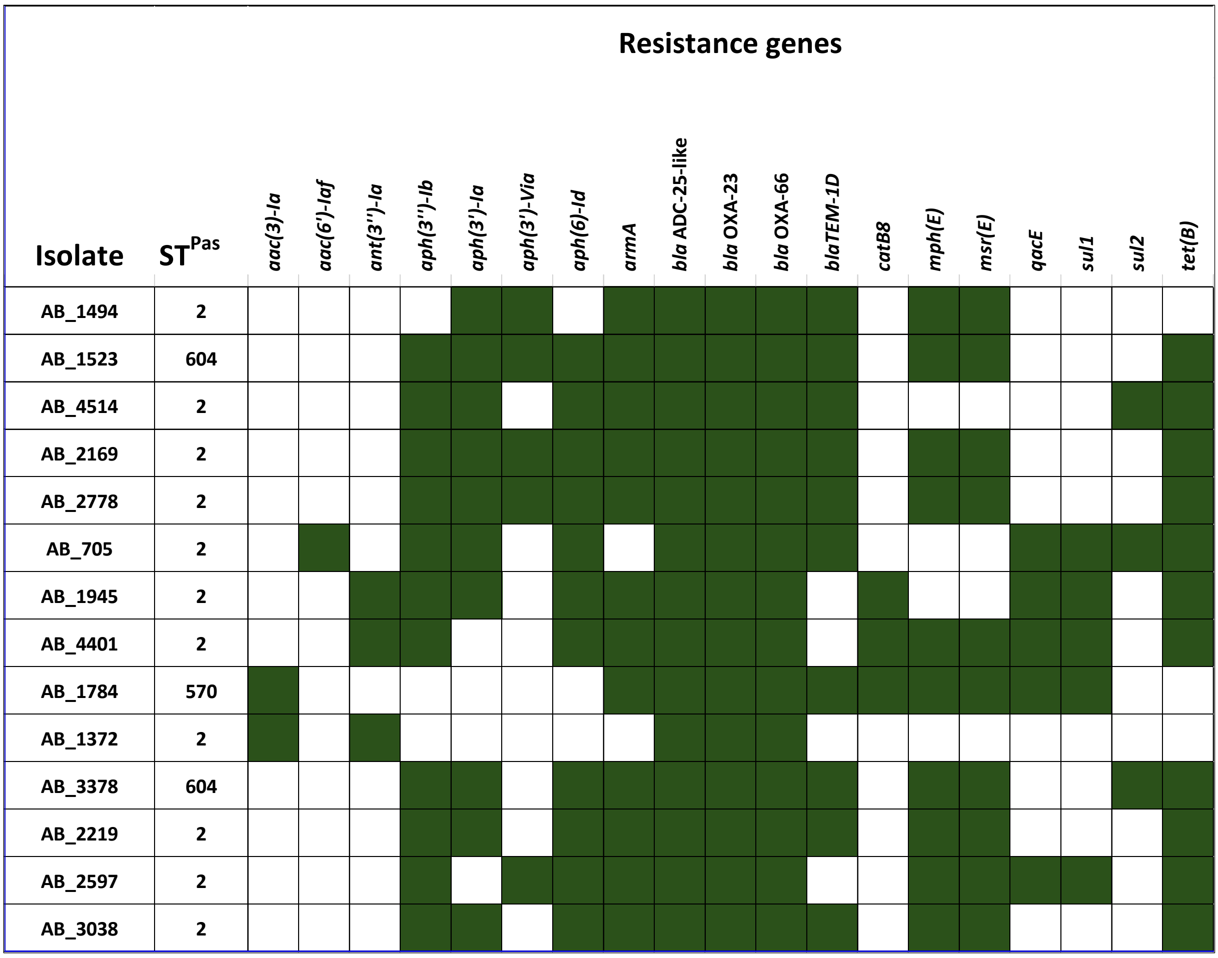

FIG S2 Presence-absence matrix of antibiotic resistance genes. Green areas indicate the presence of the resistance gene in the respective genome. ST^{Pas} = sequence type according to Pasteur scheme.

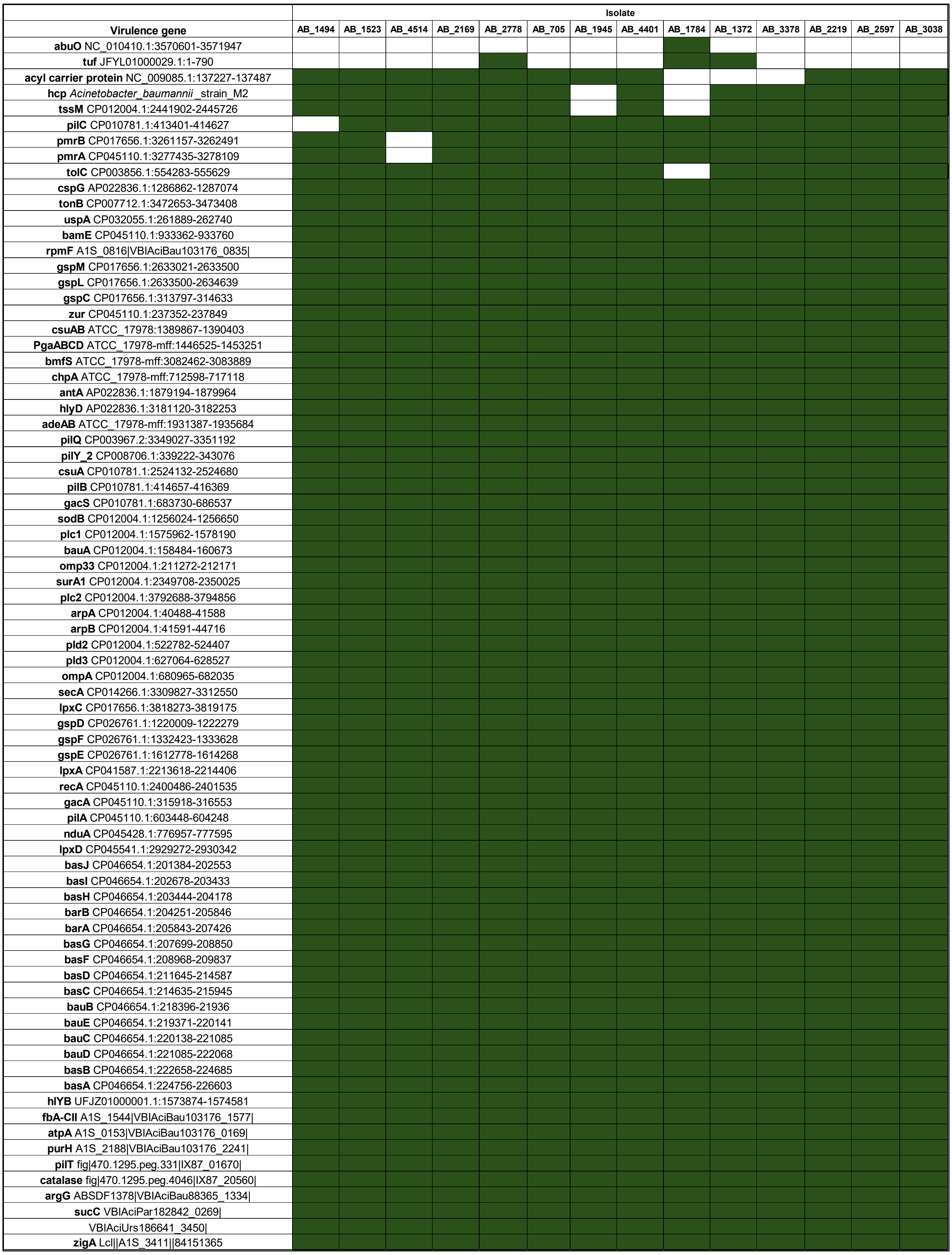

FIG S3 Presence-absence matrix of virulence genes. Green areas indicate the presence of the resistance gene in the respective genome. ST^{Pas} = sequence type according to Pasteur scheme.

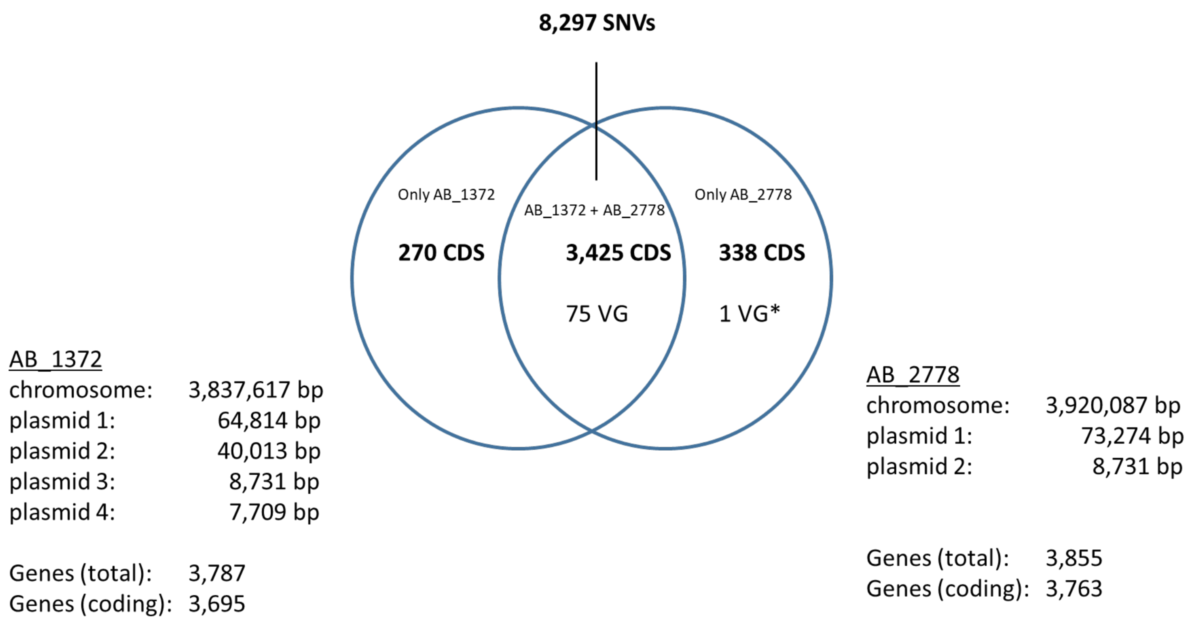

FIG S4 Comparison of genomes between AB_1372 and AB_2778. SNVs, single nucleotide variants; CDS, coding gene sequences; VG, virulence genes; VG*, acyl carrier protein NC_009085.1:137227-137487.

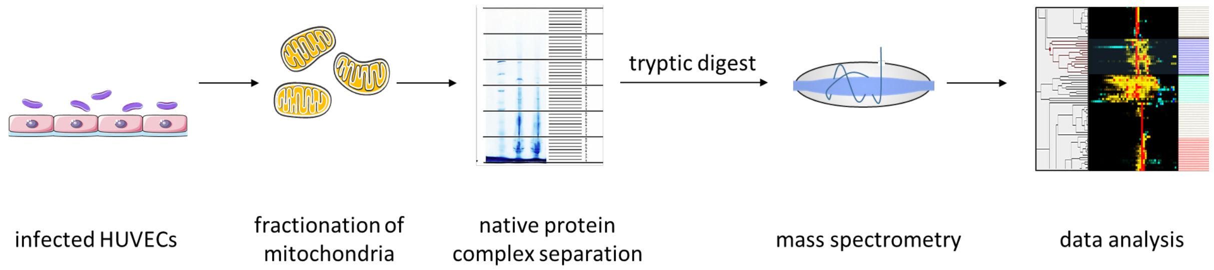

FIG S5 The workflow of the complexome analysis is depicted. Mitochondria of *A. baumannii* infected HUVECs were fractionated and proteins were separated by blue native page. The gel was sliced into 48 pieces followed by a tryptic digest and mass spectrometry. Migration profiles of identified proteins were analysed and protein complexes were clustered using NOVA.

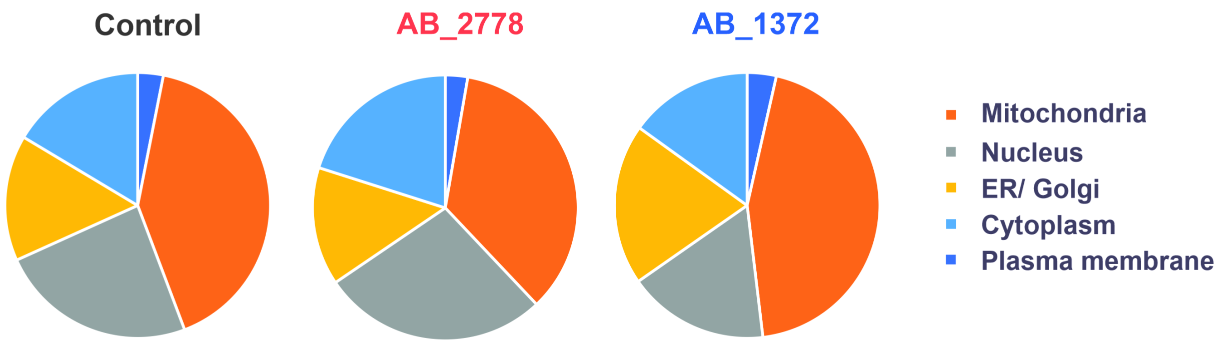

FIG S6 The gene ontology term was assigned to each protein from complexome profiling. The proportions of each cell compartment were plotted for the uninfected control and for HUVECs infected with AB_2778 and AB_1372, respectively.

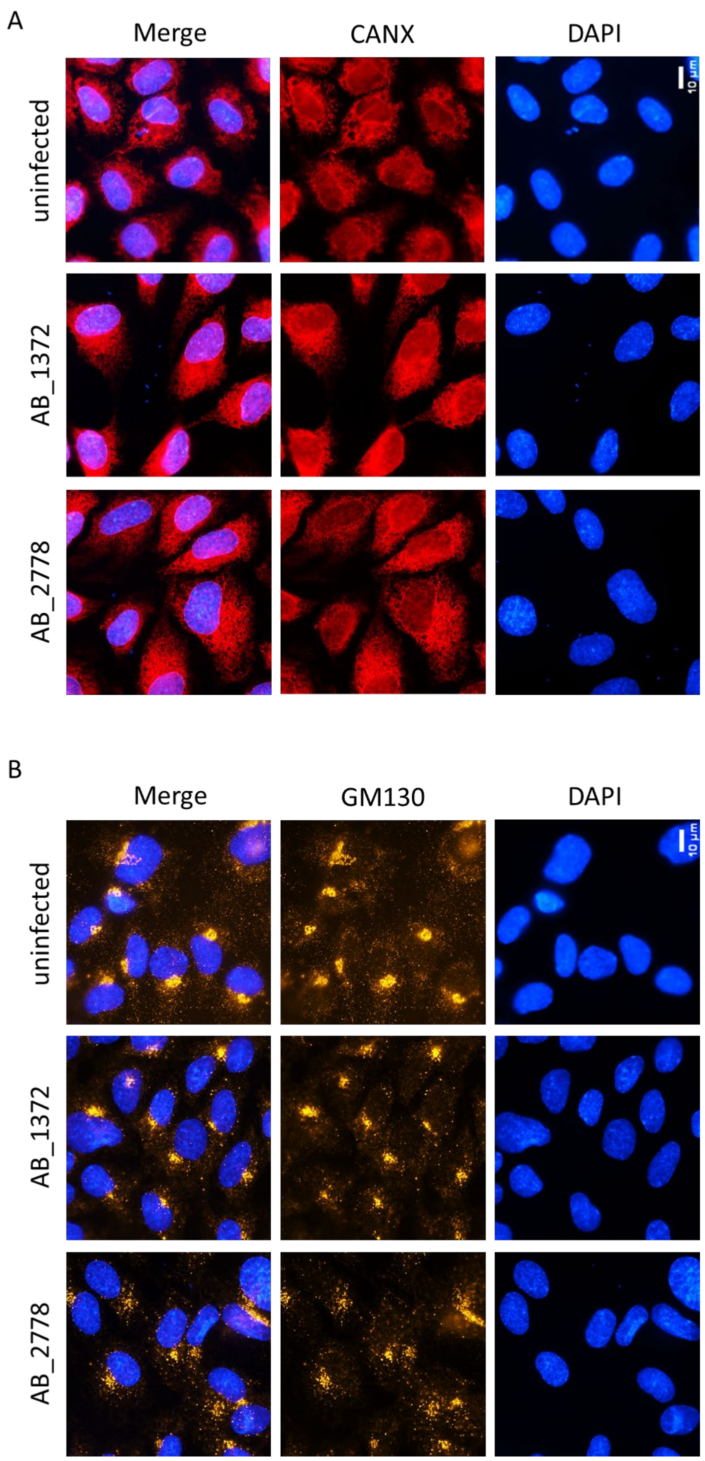

FIG S7 Analysis of morphology of endoplasmic reticulum (A) and Golgi apparatus (B) upon bacterial infection. HUVECs were infected with AB_1372 or AB_2778 for 16 hours (MOI 50). Representative immunofluorescence images of either uninfected and infected HUVECs. DAPI staining was used for visualisation of DNA (blue). The endoplasmic reticulum marker protein CANX (rot) and the Golgi apparatus marker protein GM130 (gold) were immunostained and morphology was monitored (scale bar: 10 µm).

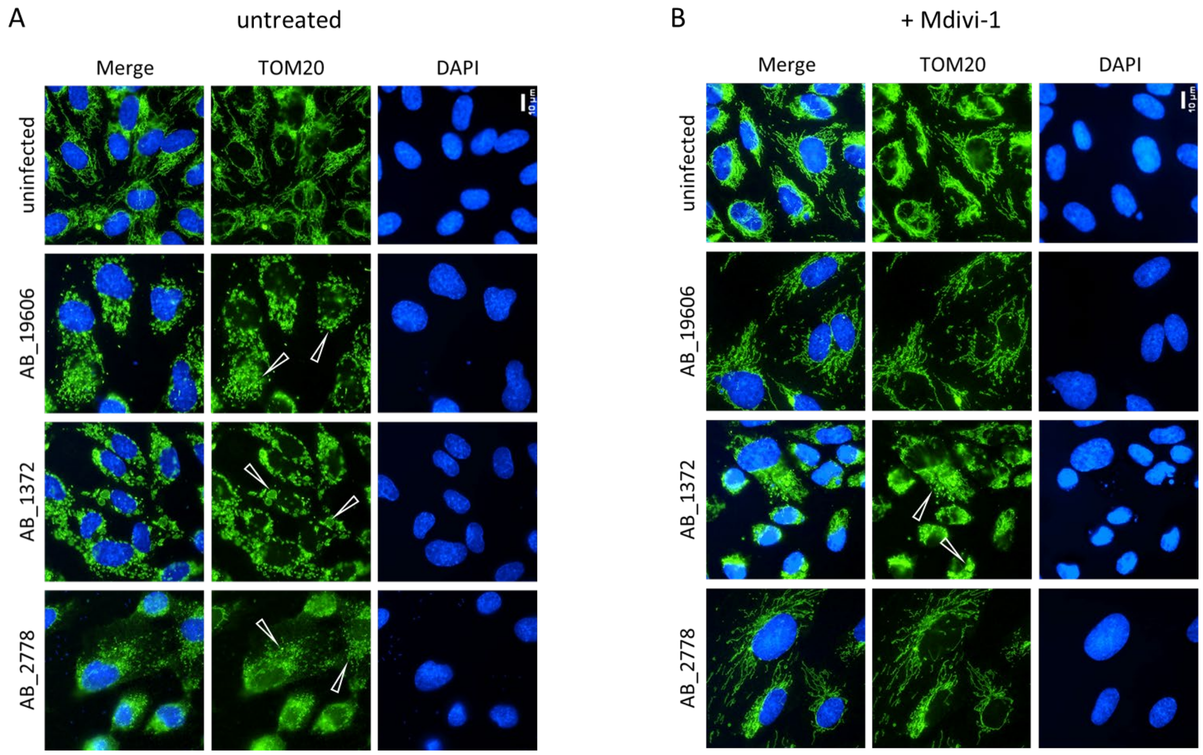

FIG S8 Impact of the inhibitor Mdivi-1 on mitochondrial morphology upon bacterial infection. (A) Pictures depict changes in morphology of mitochondria upon infection with AB_1372, AB_2778 and AB_19606, respectively, 10 hpi (MOI 50). (B) Representative immunofluorescence images of uninfected and infected HUVECs after pre-treatment with 25 µM Mdivi-1 (DRP-1 inhibitor) 14 hours prior to infection. DAPI staining was used for visualisation of DNA (blue). The outer mitochondrial membrane protein TOM20 (green) was immunostained and mitochondrial morphology and dynamics were monitored (scale bar: 10 µm). Triangles in TOM20 images indicate morphological changes of mitochondria compared to uninfected control.

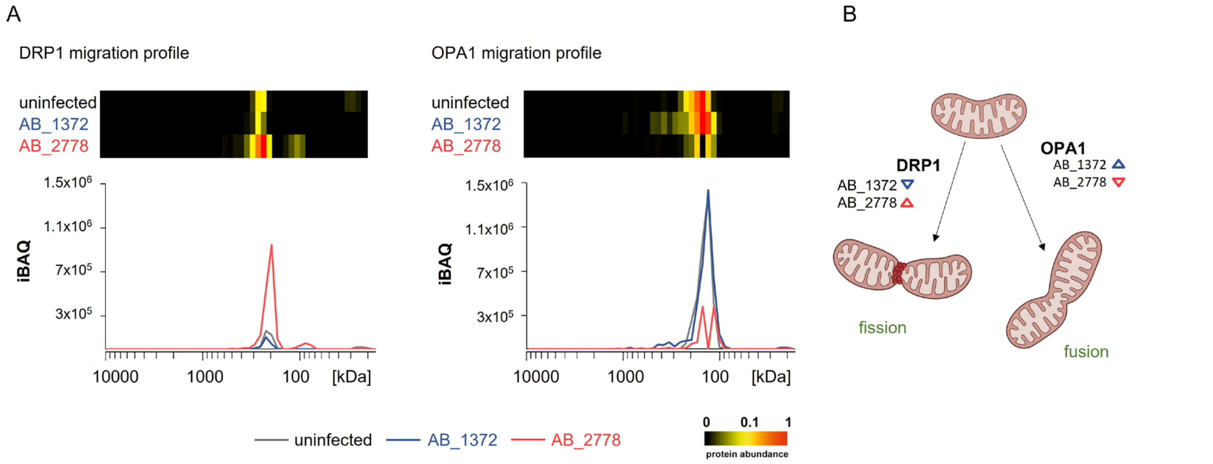

FIG S9 Abundance of DRP1 and OPA1 upon bacterial infection (A) Heat map of normalized protein abundance distribution and migration profile of absolute protein abundance of DRP1 and OPA1 upon infection with AB_1372 and AB_2778, respectively and uninfected HUVECs. (B) Direction of triangles indicate abundance of OPA1 and DRP1 in infected HUVECs for the AB_1372 and AB_2778, respectively.

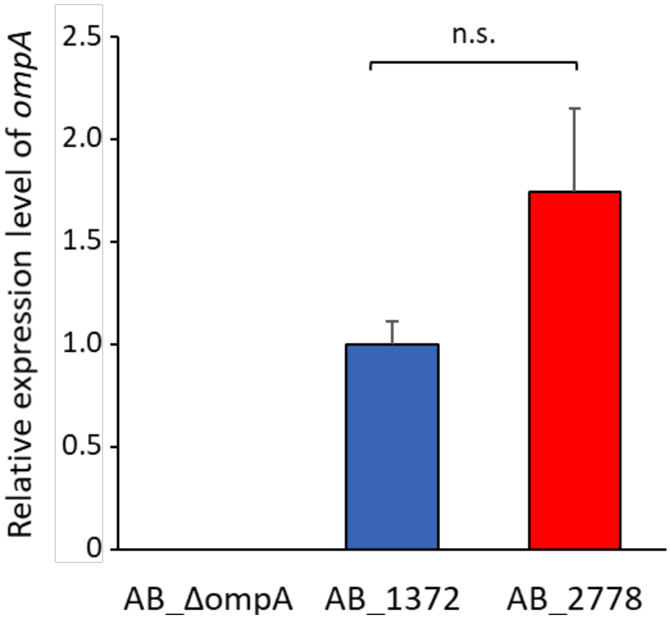

FIG S10 Relative expression levels of *ompA* in AB_1372, AB_2778 and AB_*ΔompA* (internal control) isolated from HUVECs after infection. Total RNA was isolated and relative levels of gene expression were measured by qRT-PCR using the housekeeping gene *rpoB*. The relative expression of *ompA* was normalized to AB_1372. Values are means ± SEM derived from two independent experiments with each four technical replicates. Statistical analysis was performed by a two-tailed t-test. n.s., not significant.

Supplementary Table

TABLE S1 Ratio of *in vitro* cytotoxicity and *in vivo* virulence, calculated by dividing the percentage of apoptotic HUVECs in Annexin V/PI assay and log(LD_{50}) from *G. mellonella* time-kill experiments.

Isolate	Apoptotic HUVECs[%]/log(LD_{50})	Apoptotic HUVECs[%]	Log(LD_{50})
AB_1372	0.76 ± 0.33	4.59 ± 1.22	6.05 ± 0.14
ATCC 19606^{T}	1.46 ± 0.63	7.38 ± 2.14	5.07 ± 0.18
AB_1523	2.45 ± 0.86	12.24 ± 3.24	4.99 ± 0.18
AB_3378	2.47 ± 0.20	12.78 ± 1.52	5.18 ± 0.34
AB_2597	2.68 ± 0.44	11.65 ± 2.27	4.35 ± 0.11
AB_1784	2.82 ± 0.24	10.86 ± 1.57	3.85 ± 0.20
AB_2219	2.94 ± 1.08	13.05 ± 3.73	4.43 ± 0.22
AB_4514	2.95 ± 0.26	12.03 ± 1.76	4.08 ± 0.15
AB_2169	2.98 ± 0.18	11.73 ± 1.42	3.94 ± 0.14
AB_5075	3.14 ± 0.09	17.37 ± 1.20	5.53 ± 0.19
AB_1494	3.27 ± 0.47	15.36 ± 2.67	4.70 ± 0.12
AB_4401	3.38 ± 0.03	14.47 ± 0.67	4.29 ± 0.13
AB_3038	3.81 ± 0.45	15.51 ± 2.62	4.07 ± 0.16
AB_705	3.87 ± 0.16	18.45 ± 1.65	4.77 ± 0.20
AB_1945	4.28 ± 0.32	18.02 ± 2.37	4.21 ± 0.20
AB_2778	5.50 ± 0.44	22.58 ± 3.12	4.11 ± 0.12

Supplementary methods

Genome sequencing

Library preparation for Nanopore sequencing was done using the SQK-RBK004 rapid barcoding kit. Sequencing was performed on a MinION MK1B sequencer utilizing R9.4.1 flow cells. Raw signal data was base called and demultiplexed using the high accuracy base calling model of guppy basecaller version 4.0.11. Raw data was filtered using NanoFilt for long reads resulting in datasets of reads with an average genome coverage of at least 30-fold for long reads. *De novo* hybrid assembly was conducted using Unicycler version 0.4.8 utilizing the bold assembly mode. Gene presence-absence plots were drawn utilizing a python script (accessible via https://raw.githubusercontent.com/sanger-pathogens/Roary/master/contrib/roary_plots/roary_plots.py).

Growth kinetics of *A. baumannii*

To compare growth of AB_1372 and AB_2778, bacteria cultures were adjusted to an optical density (OD_{600}) of 0.05 in lysogeny broth (LB). A total of 200 µL of the bacterial solutions were transferred to 96-well plates and incubated in a plate reader (infinite M200 Pro, TECAN) for 24 h at 37 °C. OD_{600} was measured every 20 min with a shaking amplitude of 3 mm during measurement.

Biofilm analysis

Bacteria were cultivated in tryptic soy broth (TSB) medium without dextrose at 37 °C overnight. Subsequently, a culture with TSB medium with 2% (w/v) dextrose was inoculated and cultivated until an OD_{600} of 0.5 was reached. A 1:20 dilution of the culture (or medium as negative control) was transferred into a 96-well microtitre plate (Nunclon Delta Surface, Thermo Fisher) and incubated for 24 h at 37 °C. The supernatant was discarded and the biofilm was washed three times with PBS. Subsequently, the plate was dried at 65 °C for 5 min. The biofilm was stained for two minutes using a saturated solution of crystal violet and washed five times with *A. dest*. Subsequently, ethanol (200 µl) was added to each well and absorption at 405 nm (crystal violet) and 570 nm (reference) was measured with a plate reader (infinite M200 Pro, TECAN).

Analysis of bacterial adhesion to human endothelial cells

HUVECs were seeded into 6-well plates and infected with *A. baumannii* (MOI 200). After infection, the 6-well plates were incubated for 1 h. The supernatant was removed and cells with adherent bacteria were washed with PBS and detached using a cell scraper. Thereafter, adherent bacteria were quantified by plating serial dilution series. Visualisation of bacterial

adhesion to human endothelial cells was done by fluorescence microscopy. For this purpose, HUVECs were seeded into 6-well plates containing collagenized coverslips and infected with *A. baumannii* (MOI 200) for 2 h. Before infection, bacteria were stained with carboxyfluorescein succinimidyl ester (CFSE) (diluted 1:300 in 1 mL *A. dest.*) for 30 min (37 °C). After incubation, cells were washed to remove non-adherent bacteria and fixed with 400 µL 3.75% paraformaldehyde (4 °C) for 15 min. Cells were thereafter incubated with 0.2% Triton X-100 for 15 min at room temperature. The incubation was followed by staining the cells with 500 µL tetramethylrhodamine (TRITC)-phalloidin (1:500) for 1 h at room temperature and 4',6-diamidino-2-phenylindole (DAPI) for 10 min at 4 °C. Bacterial adhesion to HUVECs was then visualised by fluorescence microscopy (Nikon Eclipse Ci-L).

Analysis of caspase activity

Activity of caspase-3, caspase-7, caspase-8 and caspase-9 were determined in 96-well plates infected with *A. baumannii* (MOI 10) using the Caspase-Glo® 3/7 Assay and the Caspase-Glo® 8 and 9 Assay (Promega, Walldorf, Germany). After infection, the 96-well plates were incubated for 2, 4, 6, 8, 16 and 24 h (37 °C, 5% CO_2). After incubation, 10 µL of the caspase-3/7 or caspase-8 or caspase-9 solution were added to each well. The plates were mixed for 30 s (300 rpm) in a plate reader (infinite M200 Pro, TECAN) and incubated for 45 min. Thereafter, plates were mixed again for 30 s (300 rpm) and luminescence was recorded.

Evaluation of bacterial virulence using the *Galleria mellonella in vivo* infection model

Larvae of the greater wax moth (*Galleria mellonella*) with a weight of 150-250 mg were obtained from UK waxworms (Sheffield, UK). *A. baumannii* isolates were grown overnight in LB and then diluted 100-fold into fresh LB and grown for 3 h. After two washes with phosphate-buffered saline (PBS), bacteria were resuspended in PBS to a final OD_{600} of 1.0 and 10-fold serial dilutions were prepared in PBS. Each experiment included control groups of non-injected larvae or larvae injected with PBS. Larvae were considered dead if they did not respond to physical stimuli. Generation of time-kill curves and calculation of the median lethal dose (LD_{50}) after 24 h was done by non-linear regression analysis using GraphPad Prism as described (49, 50, 54). Experiments were repeated three times using ten larvae per experimental group.

Determination of *ompA* expression levels

Quantification of *ompA* gene expression was done as described previously (58). Total RNA was isolated from bacteria harvested after infection of HUVECs (Monarch Total RNA Miniprep Kit, NEB, Frankfurt am Main, Germany). Subsequently, samples were used for quantitative real-time PCR (qRT-PCR). Expression levels of *rpoB* as internal control (*rpoB*_RT_fwd primer: GAG TCT AAT GGC GGT GGT TC; *rpoB*_RT-rev Primer: ATT GCT TCA TCT GCT GGT TG)

and *ompA* (*ompA*_RT_F primer: AGG TCA CAC AGA TAA CAC TGG; *ompA*_RT_R primer: AAC GTT GTA TTC GTT TAC AAG AGC) (Luna, Universal One-Step RT-qPCR Kit, NEB, Frankfurt am Main, Germany) were quantified (11). The relative expression of *ompA* in bacteria after infection was normalized to planktonic bacteria.
